# Internet Search Alters Intra- and Inter-regional Synchronization in the Temporal Gyrus

**DOI:** 10.3389/fpsyg.2018.00260

**Published:** 2018-03-06

**Authors:** Xiaoyue Liu, Xiao Lin, Ming Zheng, Yanbo Hu, Yifan Wang, Lingxiao Wang, Xiaoxia Du, Guangheng Dong

**Affiliations:** ^1^Department of Psychology, Zhejiang Normal University, Jinhua, China; ^2^Center for Life Sciences, Peking University, Beijing, China; ^3^Department of Psychology, London Metropolitan University, London, United Kingdom; ^4^Department of Physics, Shanghai Key Laboratory of Magnetic Resonance, East China Normal University, Shanghai, China; ^5^Institute of Psychological and Brain Sciences, Zhejiang Normal University, Jinhua, China

**Keywords:** internet-search, short-term training, regional homogeneity, functional connectivity, long-term memory

## Abstract

Internet search changed the way we store and recall information and possibly altered our brain functions. Previous studies suggested that Internet search facilitates the information-acquisition process. However, this process may cause individuals to lose the ability to store and recollect specific contents. Despite the numerous behavioral studies conducted in this field, little is known about the neural mechanisms underlying Internet searches. The present study explores potential brain activity changes induced by Internet search. The whole paradigm includes three phases, namely, pre-resting state fMRI (rs-fMRI) scan, 6-day Internet search training, and post rs-fMRI scan. We detected the functional integrations induced by Internet search training by comparing post- with pre-scan. Regional homogeneity (ReHo) and functional connectivity (FC) were used to detect intra- and interregional synchronized activity in 42 university students. Compared with pre-scan, post-scan showed decreased ReHo in the temporal gyrus, the middle frontal gyrus, and the postcentral gyrus. Further seed-based FC analysis showed that the temporal gyrus exhibited decreased FC in the parahippocampal cortex and the temporal gyrus after training. Based on the features of current task and functions exhibited by these brain regions, results indicate that short-term Internet search training changed the brain regional activities involved in memory retrieval. In general, this study provides evidence that supports the idea that Internet search can affect our brain functions.

## Introduction

Finding information through Internet search engines has become a common daily activity for people (Small et al., 2009). The widespread use of the Internet changed the way we find and store information. “Google effect” indicates that when people use the Internet as an external storage, they need to remember “where” it is instead of the information itself (Sparrow et al., 2011; Ward, 2013). These studies suggested that Internet search reduced the need for effort to process and remember information (Carr, 2010).

Recent studies explored the influence of Internet search on the brain. Nicholas suggested that the younger “Google generation” spends less time on individual questions and searches quicker, but the members of this generation show poorer working memory and are less confident about the answers they provided compared with the older generation (Nicholas, 2013). Small et al. (2009) showed that prior experience of using Internet search increased brain responsiveness in neural circuits involved in decision-making and complex reasoning in aged adults after short-term training. The brain activities of experienced Google users were broader than that of novices during searches (Small et al., 2009). A previous study found that people who obtained information through Internet-based search memory task would show lower accuracy when recalling information and are highly impulsive in novel trials (Dong and Potenza, 2015). However, 6 days of practicing Internet search improved their efficiency, but it reduced their dependency on their long-term memory (Dong and Potenza, 2016; Dong et al., 2017).

Brain and behavior are a dynamic system that influences each other and form the basis of brain plasticity (Mechelli et al., 2004). A large number of studies proved that learning induced brain plasticity (Kolb and Whishaw, 2003; Vartanian et al., 2013). In addition, various tasks generated differentiated functional response patterns through modulation of training (Koeneke et al., 2004; Kelly and Garavan, 2005; Erickson et al., 2007; Thomas et al., 2009). Previous studies revealed that 3 months of juggling training could lead to a transient bilateral expansion in gray matter in the mid-temporal area and in the left posterior intraparietal sulcus (Draganski et al., 2004). Five hours of meditation training can change the activity of default mode network and connectivity of the white matter (Brewer et al., 2011; Ding et al., 2015). The neural circuitry of the frontal pole, anterior temporal cortex, and anterior and posterior cingulate was activated in Internet-savvy subjects after 5 days of practice (Small et al., 2009).

Researchers recently realized that the effect of training/expertise-specific on brain functions may extend beyond task state to resting state. Resting state refers to the state when subjects relax, stationary, eyes closed, and avoid any systematic thinking (Mazoyer et al., 2001). Resting-state fMRI was also used to study intrinsic functional connectivity (FC) and has been widely used as a tool to assess large-scale networks in the human brain in both clinical and healthy populations (Wink et al., 2006; Tian et al., 2007; Buckner et al., 2013).

Regional homogeneity (ReHo) and FC are often used to evaluate brain activity synchronization in resting-state of healthy subjects and patients. ReHo is a rank-based non-parametric data-driven approach that reflects the temporal homogeneity of the regional BOLD signal (Sepulcre et al., 2010). ReHo measures the functional coherence of a given voxel with its nearest neighbors by calculating Kendall’s coefficient concordance (KCC) (Zang et al., 2004; Zuo et al., 2012). The test–retest reliability of ReHo has been found very high despite the physiological noise and preprocessing effect (Zuo et al., 2012; Zuo and Xing, 2014). FC measures the similarity of the time series of two relatively remote brain regions (Biswal et al., 1995). These two measures are often used together to detect local and remote brain activity synchronizations. Hence, combining the ReHo and FC analysis could provide additional information about brain activity synchronization induced by Internet search.

Numerous studies revealed that short-term training could alter the resting-state features of our brain. An existing study found resting-state coherence in the right medial motor cortex was increased by brief sensory motor intervention (Verrel et al., 2015). A study on acupuncturists found that training/expertise could modulate resting-state activity by increasing regional clustering strength (Dong et al., 2014). In a short-term simulated microgravity study, 72 h of -6° head down tilt (HDT) resulted in decreased ReHo in the right inferior frontal gyrus (Liao et al., 2013). Modulation of resting-state functional connectivity (rs-FC) in the parietal circuit was found after 4 weeks of daily training of an explicit sequence learning task (Ma et al., 2011). A study found that 4 weeks of working memory training increased rs-FC between the medial prefrontal cortex (mPFC) and the precuneus, but decreased rs-FC between the mPFC and the right posterior parietal cortex (Takeuchi et al., 2013). These studies suggest that the resting-state features of the brain could be altered by short-term training.

Given these findings, we speculated that the brain activity in resting-state would be affected by search engines usage. In the present study, we first explore abnormal brain activity using ReHo analysis and investigate the FC between regions with altered ReHo and other brain regions. Previous studies found that people using Internet search as a tool for remembering new information showed lower brain activations in the middle temporal gyrus (Dong and Potenza, 2015) and regions along the ventral stream (Knutson et al., 2012). In addition, people using Internet search tools just need to remember where the information is stored instead of the information itself (Sparrow et al., 2011). Hence, we hypothesized that short-term training would make people to be better at remembering where information is stored (higher brain activities in regions along the dorsal stream) than the specific content (lower brain activities in regions along the ventral stream). We compared data on resting brain states between pre- and post-scan from 42 college student volunteers to examine the changes of brain activity induced by Internet search by measuring ReHo and FC.

## Method and Procedure

### Participants

The experiment complied with the Code of Ethics of the World Medical Association (Declaration of Helsinki). The Human Investigations Committee of Zhejiang Normal University approved this study. This study was conducted in accordance with the approved guidelines. Forty-two university students were recruited through advertisements (22 males; 20 females; age: 21.4 ± 1.2 years). All participants provided written informed consent and underwent structured psychiatric interviews using the Mini-International Neuropsychiatric Interview (MINI) (Lecrubier et al., 1997) performed by an experienced psychiatrist. According to the MINI assessment, they were free of psychiatric disorders, including major depression, anxiety disorders, schizophrenia, and substance dependence disorders. All participants were medication-free and were instructed not to use any substances, including coffee, on the day of scanning. To obtain information regarding their Internet search behaviors, all subjects were assessed using an Internet-search-use questionnaire (Supplementary Material) (Dong and Potenza, 2015; Wang et al., 2016). Results showed that all participants were familiar with Internet search and used it regularly. We included Internet search experience as a covariate to exclude the impact on experimental results (Wang et al., 2016).

### Experiment Procedure

The whole experiment consists of three steps, namely, pre-scan, 6 days of training, and post-scan.

Subjects were “trained” for at least 1 h per day for six consecutive days. In the experiment, subjects were asked to finish one of six search tasks randomly without repetition. Each search task consisted of 80 fill-in-the-blank items that required subjects to seek answers using an Internet search engine. Participants were informed that they will receive 20 Chinese yuan for their everyday participation. To elicit motivation in searching, the subjects were advised that the daily reward would be paid based on their real performance [20 ^∗^ accuracy rates (%)]. This reward premise was approved by the ethics committee. Participants who took the work seriously and finished with an accuracy rate of over 80% passed the training.

### Image Acquisition

Functional MRI was performed on a 3.0 Tesla Siemens Trio scanner. The functional scan was acquired using gradient echo planar imaging sequence with the following parameters: [repeat time (TR) = 2 s, echo time (TE) = 30 ms, flip angle = 90°; interleaved sequence; 33 slice per volume; 3 mm thickness; field of view (FOV) = 220 mm × 220 mm^2^, matrix 64 cm × 64 cm, acquisition matrix = 64 × 64]. Each functional run included 210 imaging volumes for each participant and the scan lasted 7 min. All subjects were instructed to rest quietly in the scanner without falling asleep. Post-scan conforms to the same standard and parameter.

### Data Pre-processing

Data pre-processing was conducted using Data Processing Assistant for Resting-State fMRI (DPARSFA^1^), a MATLAB toolbox for “pipeline” data analysis of resting-state fMRI (Yan and Zang, 2010; Song et al., 2011). DPARSFA is based on some functions in Statistical Parametric Mapping^2^ and Resting-State (REST^3^). The main preprocessing steps and parameters are listed as follows. The first 10 volumes of each functional time series were abandoned to avoid the instability of the initial fMRI signal, thereby leaving 200 volumes. Preprocessing included slice timing, head motion correction, and spatial normalization to a standard template. Participants with maximum translation that exceeds 2.5 mm or maximum rotation that exceeds 2.5° were excluded from further analysis. To reduce the effects of confounding factors, a regression of nuisance signals including cerebral spinal fluid, white matter, six motion vectors was performed. Following regression, detrending was performed and temporal filtering (0.01 to 0.08 Hz) was applied to the time series of each voxel to reduce low-frequency drift and high-frequency noise. Data for one subject were excluded according to the head-motion parameter (2.5 mm; 2.5°).

### ReHo Analysis

Resting-state fMRI data without spatial smoothing were used for ReHo analysis with DPARSFA. Individual ReHo maps were generated by calculating the KCC of the time series of a given voxel with its nearest neighbors (26 voxels) in all directions on a voxel-wise basis. The calculated formula of ReHo is defined as follows:

$$\begin{array}{c} W=\frac{\sum {\left(R_{i}\right)}^{2}-n{\left(\bar{R}\right)}^{2}}{\frac{1}{12}K^{2\;}(n^{3}-n)}, \end{array}$$

where *W* is the KCC for a given voxel that ranged from 0 to 1. *R*_i_ is sum rank of the *i*th time point and *n* is the number of ranks; $\bar{R}$ = [(*n*++1) K]/2 is the mean of the *R*_i_; *K* is the voxel number among time series (27 voxels, one given voxel plus the number of its neighbors). To reduce the influence of individual variations in the KCC value, each standardized ReHo map was generated by dividing the raw ReHo map by the global mean ReHo. Spatial smoothing was conducted on the ReHo maps with a Gaussian kernel of 4 mm × 4 mm × 4mm full-width at half-maximum (Liao et al., 2013; Chen et al., 2015).

### FC Analysis

A seed-based correlation approach was used for FC analysis. The seed was defined in the regions’ existing difference and analyzed by ReHo. We calculated the temporal correlation between these seed regions and every other voxel within the brain. These procedures were executed using DPARSFA software.

### Post–Pre Analysis

To explore the differences between the pre- and post-training, a pair-sample *t*-test was performed on the normalized ReHo and FC maps with REST software. The result and statistical map were set at a combined threshold of *p* < 0.05 (AlphaSim corrected) and a minimum cluster size of 110 voxels.

## Results

### Regional Homogeneity (ReHo)

**FIGURE 1:**
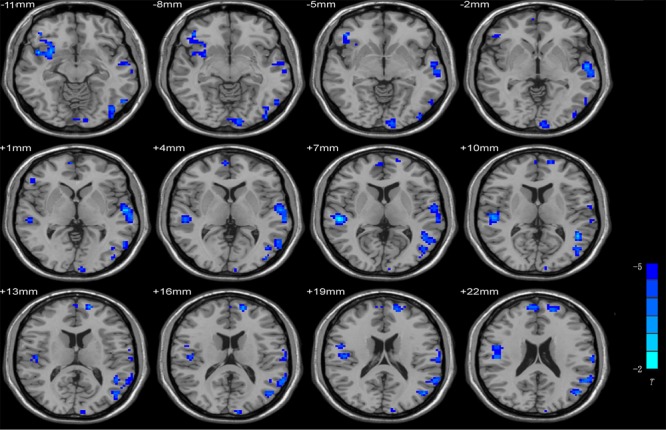
Brain areas with increased and decreased ReHo in post-scan compared with pre-scan. Pair-sample *t*-test *p* < 0.05, AlphaSim corrected, voxel size = 3 × 3 × 3; *T*-score bars are shown on the right bottom. The voxels with hot colors represent increased ReHo after training, and cold colors indicate decreased ReHo after training.

Compared with the pre-scan data, the neuroimaging data from post-scan were associated with the decreased ReHo values in the temporal gyrus, which include the superior temporal gyrus and the middle temporal gyrus, the middle frontal gyrus and the postcentral gyrus. **Table 1** shows the detailed information for the brain regions with ReHo difference after training. **Figure 1** shows the brain areas.

**Table 1:** Brain areas showing Regional homogeneity (ReHo) difference between post- and pre-training.

Areas	Hemisphere	Voxels	Peak	Coordinates
			*t*-value	
**Decreased ReHo after training**				
Superior temporal gyrus	R	147	–5.3319	54–24 6
Middle temporal gyrus	L	157	–4.7822	–48–72 18
Middle frontal gyrus	L	345	–4.7366	–33–6 63
Postcentral gyrus	L	211	–4.4208	–60–12 0

*Voxel size = 3 × mm3 mm × 3 mm, p < 0.05 AlphaSim corrected and at least 110 voxels. L, left; R, right; t: t-values from a paired two tailed t-test of the statistical different clusters.*

### Seed-Based FC of Altered ReHo Regions

**FIGURE 2:**
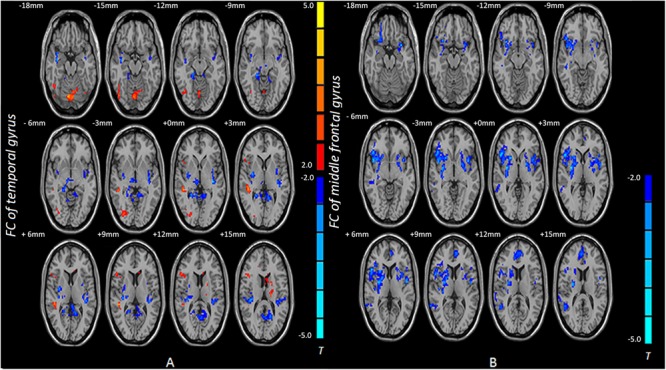
Brain areas with increased and decreased FC in post-scan compared with pre-scan. **(A)** The voxels with hot colors represent increased FC between the temporal gyrus and the parietal lobe after training, and cold colors indicate that the temporal gyrus shows decreased FC with the parahippocampal cortex and the temporal gyrus after training. **(B)** The middle frontal gyrus exhibited decreased FC with the middle temporal gyrus and the middle frontal gyrus after training. Pair-sample *t*-test *p* < 0.05, AlphaSim corrected; FC, functional connectivity.

Based on the ReHo results, the temporal gyrus and the middle frontal gyrus were selected as seed regions of interest for FC analysis. The temporal gyrus exhibited decreased FC with the parahippocampal cortex and the temporal gyrus after training whereas the temporal gyrus showed increased FC with the parietal gyrus. The middle frontal gyrus exhibited decreased FC with the middle temporal gyrus and the middle frontal gyrus after training (**Table 2** and **Figure 2**).

**Table 2:** Pre–post differences in seed-based FC in altered ReHo regions.

Areas	Hemisphere	Voxels	Peak	Coordinates
			*t*-value	
**Post-training – pre-training**				
**FC of R Temporal gyrus**				
Parietal gyrus	R	110	3.2562	36–57 45
Temporal gyrus	R	406	–4.2347	39 6–21
Parahippocampal cortex	R	446	–3.8151	24–45 0
Superior frontal gyrus	R	548	–4.9860	9–18 78
**FC of L Middle frontal gyrus**				
Superior temporal gyrus	L	374	–4.0656	–33 9–18
Middle temporal gyrus	R	417	–3.8514	42–57 60
Middle frontal gyrus	R	147	–3.4775	45 6 51

*Voxel size = 3 mm × 3 mm × 3 mm, p < 0.05 AlphaSim corrected and at least 110 voxels. L, left; R, right; FC, functional connectivity; t: *t*-values from a paired two tailed *t*-test of the statistical different clusters.*

## Discussion

This study finds decreased ReHo in the temporal gyrus, the middle frontal gyrus and the postcentral after training. The temporal gyrus exhibited decreased FC with the parahippocampal cortex and the temporal gyrus after training, whereas the temporal gyrus showed increased FC with the parietal gyrus. The middle frontal gyrus exhibited decreased FC with the middle temporal gyrus and the middle frontal gyrus after training. This evidence of local functional homogeneity and interregional FC contributes to our understanding of the changed brain activity produced by Internet search.

The middle temporal gyrus located at the end of the ventral stream, which was described as the “what” stream, is involved in object identification and recognition (Mishkin and Ungerleider, 1982; Goodale and Milner, 1992). The Decreased ReHo and FC in the temporal gyrus supports our hypothesis that short-term training decreases brain activities in regions along the ventral stream. The parahippocampal cortex is a part of the limbic system, which plays an important role in memory encoding and retrieval (Ekstrom and Bookheimer, 2007). Evidence from patient studies suggested that the middle temporal gyrus is associated with long-term memory (Squire and Zolamorgan, 1991; Meulemans and Van der Linden, 2003). Functional neuroimaging study also found that the middle temporal gyrus is activated when individuals participate in memory encoding and retrieval processes of memory (Onoda et al., 2009). Previous studies found that Internet search decreases brain activation in the temporal gyrus (Dong and Potenza, 2016). Decreased ReHo and FC in the temporal gyrus might suggest that Internet search causes people to rely less on their long-term memory.

Results showed that short-term training increased FC between the temporal gyrus and the parietal lobe. The posterior parietal cortex is referred to as the dorsal stream or “where” stream, which is involved in visuospatial processing (Mishkin and Ungerleider, 1982). Therefore, increased FC might suggest that Internet search enhances the spatial information processing (Dong et al., 2017). The result is consistent with our hypothesis. The middle frontal gyrus plays an important role in selective attention, executive control, and working memory (Fuster, 2002). Decreased ReHo and FC in the middle frontal gyrus may suggest that individuals engage less in remembering something when faced with information that can be found in the Internet. According to Sparrow, people who used Internet search engines to access information show worse recall rates of information (Sparrow et al., 2011). Our results indicated that short-term Internet search training could alter the memory system, which further provided evidence for the hypothesis that Internet search enables people to be better at remembering where information is stored than the specific content.

As the Internet evolved into a useful tool in our daily life, people become susceptible to the unprecedented Internet search environment (Zickuhr and Madden, 2012; Loh and Kanai, 2016). Some researchers found that many people are increasingly relying on Internet search (Zickuhr and Madden, 2012; Yang et al., 2014; Wang Y. et al., 2017). People show irritability and depression when they cannot immediately find what they want (Block, 2008). These symptoms are similar with withdrawal symptoms of pathological Internet use (Davis, 2001). The executive function of Internet addiction subjects is impaired, whereas the inhibition control of Internet-using behaviors of those people is weakened (Wang L. et al., 2017). Our study found that the brain activities of Internet search users behaved differently. These people might engage in less effort in remembering something after short-term training.

## Conclusion

This study found that the aberrant ReHo and FC were mainly distributed in the temporal gyrus and the middle frontal gyrus, which are responsible for long-term memory. Results provide evidence that support the idea that Internet search can affect our brain functions.

### Limitations

Several limitations should be addressed. First, given financial and time constraints, we canceled the control group and pre–post resting-state data were not collected from the control group. We paid more attention to pre–post difference in one group of subjects. Second, we found it impossible to recruit university students that have no experience in Internet search. Thus, all subjects were familiar with Internet search. This sample group might affect training effect. Given the limitation of rs-fMRI studies, the explanations of the results rely on the brain functions of relevant brain regions, which lack direct support from behavioral or task data. Thus, the explanations are based on reasoning and the interpretation of the results is uncertain. However, the findings provide evidence that Internet search can affect our brain functions.

## Author Contributions

XLiu and XLin analyzed the data and wrote the first draft of the manuscript. LW and YW contributed to experimental programming and data collection. XD contributed to fMRI data collection. GD and YH designed this research. GD and MZ revised and improved the manuscript. All authors contributed to and had approved the final manuscript.

## Conflict of Interest Statement

The authors declare that the research was conducted in the absence of any commercial or financial relationships that could be construed as a potential conflict of interest.

## Footnotes

1 www.restfmri.net

2 http://www.fil.ion.ucl.ac.uk/spm

3 http://www.restfmri.net/forum/
