# Supplementary material for: Internet Search Alters Intra- and Inter-regional Synchronization in the Temporal Gyrus
Source: Front Psychol. 2018 Mar 6;9:260. doi: 10.3389/fpsyg.2018.00260 (PMC5845706; doi:10.3389/fpsyg.2018.00260)
Supplement: Supplementary file 1 [file Data_Sheet_1.PDF]

The Questionnaire on Internet Search Dependence

Hello, we are researchers in the Department of Psychology of Zhejiang Normal University. We carry this investigation to explore how you search the Internet. This result will not be open to the public; please answer the questions honestly and seriously.

Please read the following information firstly:

1. Internet search means individuals using Internet search engines (for example, Google, Baidu) to find information on the Internet.
2. The apparatus where people use Internet search engines include computers, IPADs, smart phones and other terminal sets.

Basic information:

1. Gender: Male   Female
2. Age:
3: Grade:
4. Major:
5. Years from your first Internet use:
6. Years from your first Internet searching use:
7. The time you spend on Internet searching every day: minutes
8. Please estimate the times you search the Internet everyday:
9. The percentage of the time spent on using search engine takes____% of the total time online.

Please provide your answers according to the following criteria:

0. Never   1. Seldom   2. Sometimes   3. Usually   4. Always

1. When facing with debatable questions, I prefer to search Google/Baidu first.
2. I won’t take notes if I know I can search for the information on the Internet.
3. When I am asked a complex question, I usually try to abstract the key words from it.
4. When someone disagrees with my points, I usually search the Internet to provide the answer.
5. I think it is not necessary to remember a thing if we can find it from Internet searches.
6. When somebody asks me a question, I will search the Internet for answers if I cannot figure it out immediately.
7. I can abstract keywords quickly from a sentence for doing a potential Internet search.
8. I will be upset if I cannot find a complex question through an Internet search.
9. I think Internet search can satisfy daily needs, including learning and living.
10. I usually start the search page unconsciously when I am idle.
11. I am not confident about the answers in my memory if I cannot double-check the answers through Internet searches.
12. I think we can find reliable information through Internet searching.

Thanks for your sincerely participation!

The Dimensions of the Questionnaire on Internet Search Dependence

Habitual Internet search using:

1. When facing with debatable questions, I prefer to search Google/Baidu first.

3. When I am asked a complex question, I usually try to abstract the key words from it.

4. When someone disagrees with my points, I usually search the Internet to provide the answer.

6. When somebody asks me a question, I will search the Internet for answers if I cannot figure it out immediately.

7. I can abstract keywords quickly from a sentence for doing a potential Internet search.

Withdrawal reaction:

8. I will be upset if I cannot find a complex question through an Internet search.

11. I am not confident about the answers in my memory if I cannot double-check the answers through Internet searches.

Internet search trust:

9. I think Internet search can satisfy daily needs, including learning and living.

10. I usually start the search page unconsciously when I am idle.

12. I think we can find reliable information through Internet searching.

External storage under Internet search:

2. I won’t take notes if I know I can search for the information on the Internet.

5. I think it is not necessary to remember a thing if we can find it from Internet searches.
